# Structural Basis of Competitive Recognition of p53 and MDM2 by HAUSP/USP7: Implications for the Regulation of the p53–MDM2 Pathway

**DOI:** 10.1371/journal.pbio.0040027

**Published:** 2006-01-17

**Authors:** Min Hu, Lichuan Gu, Muyang Li, Philip D Jeffrey, Wei Gu, Yigong Shi

**Affiliations:** **1**Department of Molecular Biology, Lewis Thomas Laboratory, Princeton University, Princeton, New Jersey, United States of America; **2**Institute for Cancer Genetics, Department of Pathology, Columbia University College of Physicians and Surgeons, New York, New York, United States of America; Brandeis UniversityUnited States of America

## Abstract

Herpesvirus-associated ubiquitin-specific protease (HAUSP, also known as USP7), a deubiquitylating enzyme of the ubiquitin-specific processing protease family, specifically deubiquitylates both p53 and MDM2, hence playing an important yet enigmatic role in the p53–MDM2 pathway. Here we demonstrate that both p53 and MDM2 specifically recognize the N-terminal tumor necrosis factor–receptor associated factor (TRAF)–like domain of HAUSP in a mutually exclusive manner. HAUSP preferentially forms a stable HAUSP–MDM2 complex even in the presence of excess p53. The HAUSP-binding elements were mapped to a peptide fragment in the carboxy-terminus of p53 and to a short-peptide region preceding the acidic domain of MDM2. The crystal structures of the HAUSP TRAF-like domain in complex with p53 and MDM2 peptides, determined at 2.3-Å and 1.7-Å resolutions, respectively, reveal that the MDM2 peptide recognizes the same surface groove in HAUSP as that recognized by p53 but mediates more extensive interactions. Structural comparison led to the identification of a consensus peptide-recognition sequence by HAUSP. These results, together with the structure of a combined substrate-binding-and-deubiquitylation domain of HAUSP, provide important insights into regulation of the p53–MDM2 pathway by HAUSP.

## Abstract 2

HAUSP specifically deubiquitinates both p53 and MDM2, to unknown effect. Structural and biochemical analysis shows that HAUSP binds these targets in a mutually exclusive manner, binding MDM2 preferentially.

## Introduction

The p53 tumor-suppressor protein is a sequence-specific transcription factor that responds to a wide variety of cellular stress signals [1–3]. The normal function of p53 is indispensable to many cellular processes, such as cell-cycle control and apoptosis. It is generally believed that p53 activity is primarily controlled at the level of post-translational modification, particularly ubiquitylation [4–6]. In normal cells, p53 is maintained at a low level mainly through MDM2-mediated ubiquitylation and subsequent degradation [7–9]. MDM2 is a RING-finger E3 ubiquitin ligase [10] that specifically interacts with p53 through its N-terminal p53-binding domain [11–14]. The C-terminal RING-finger motif of MDM2 not only promotes p53 ubiquitylation [10,15], but also targets MDM2 itself for auto-ubiquitylation and subsequent degradation [10,15]. In addition, MDM2 is also a negative regulator of p53-mediated transcriptional activity [16]. The MDM2 gene is a transcriptional target of p53 [17–19], so activation of the MDM2 gene by p53 would lead to repression of p53 activity. The existence of this auto-regulatory feedback loop between MDM2 and p53 adds a complex feature to the p53–MDM2 pathway and makes MDM2 one of the most important regulators of p53 activity.

As a countermeasure of protein ubiquitylation, protein deubiquitylation has also been recognized as an important regulatory step in many cellular processes [20–23]. Most deubiquitylating enzymes (DUBs) are cysteine proteases that specifically cleave ubiquitin from its conjugates. Growing evidence shows that DUBs can act at many different stages throughout the ubiquitin–proteasome pathway and thus play a key role in maintaining the normal functions of the ubiquitin-dependent system. There are five conserved families of DUBs, of which the ubiquitin-specific processing proteases (UBPs) constitute the largest family with more than 60 members [20–23]. A unique feature for UBPs is that they contain divergent N- and/or C- terminal extensions, suggesting putative substrate-recognition modules. However, only a few UBPs with specific substrates have been identified and, consequently, the mechanisms by which UBPs recognize substrates remain largely unknown.

Using an affinity-based approach, herpesvirus-associated ubiquitin-specific protease (HAUSP, also known as USP7) was identified as a novel p53-interacting protein [24,25]. HAUSP directly binds to and deubiquitylates p53 both in vivo and in vitro. Expression of HAUSP was shown to stabilize p53 in vivo and to promote p53-dependent apoptosis and cell-growth arrest [24]. These observations revealed an important mechanism for p53 stabilization and identified HAUSP as the first mammalian UBP with a known substrate. More recently, HAUSP was shown to physically interact with MDM2 in a p53-independent manner [26–28]. HAUSP can deubiquitylate MDM2, both in vivo and in vitro, and is required for the stability of MDM2 in normal cells [26–28].

The observation that HAUSP can directly interact with and deubiquitylate both p53 and MDM2 creates a conundrum: how can HAUSP stabilize p53 while at the same time being able to stabilize MDM2, which is primarily responsible for the destruction of p53? One possibility is that only one of the two proteins, p53 and MDM2, represents a physiologically relevant target for HAUSP [27,28]. Supporting this notion, increased ubiquitylation of MDM2 was observed in the HAUSP-ablated cells, which consequently resulted in the stabilization of p53 [26–28]. Another contrasting possibility is that HAUSP can target both p53 and MDM2 for deubiquitylation and thus may play a dynamic role in the p53–MDM2 pathway [26]. In both scenarios, in order to decipher the role of HAUSP in the p53–MDM2 pathway, it is essential to understand how HAUSP specifically recognizes p53 and MDM2 and to understand the relationships involved in these two recognition events.

The N-terminal tumor necrosis factor–receptor associated factor (TRAF)–like domain (residues 53–208) of HAUSP was previously shown to specifically interact with the C-terminal sequences of p53 [29]. A recent paper reported the crystal structures of the TRAF-like domain of HAUSP in isolation and in complex with a peptide derived from EBNA1 [30]. Although the structure of the HAUSP TRAF-like domain closely resembles that of other known TRAF domains, recognition of the EBNA1 peptide by HAUSP is different from that observed in other TRAF–peptide complexes [30]. Interestingly, analysis of the nuclear magnetic resonance chemical shifts suggested that a p53 peptide might bind to the same general location on the surface of the HAUSP TRAF-like domain as the peptide derived from EBNA1 [30].

In this paper, we report the biochemical and structural basis on the recognition of p53 and MDM2 by HAUSP. We show that both p53 and MDM2 bind to, in a mutually exclusive manner, the N-terminal TRAF-like domain of HAUSP. MDM2 exhibits a much higher binding affinity, as it efficiently out-competes excess p53 for binding to HAUSP in a competition assay. We localized the HAUSP-binding elements to short-peptide fragments in p53 and in MDM2 and determined the crystal structures of HAUSP TRAF-like domain bound to such peptides. These structures reveal the molecular basis for the differential binding of p53 and MDM2 to HAUSP, which allows the derivation of a consensus peptide-recognition sequence for HAUSP. These findings have important ramifications for understanding the role of HAUSP in the p53–MDM2 pathway.

## Results

### Structure of the HAUSP TRAF-Like Domain

We previously mapped the p53-binding element of HAUSP to its N-terminal residues 53–208 [29], which shares significant homology to the TRAF domain [31]. We crystallized this domain (residues 53–206) and determined its structure at 1.65-Å resolution (Tables 1–3; Figure 1A). As previously reported [30], the HAUSP TRAF-like domain adopts an eight-stranded anti-parallel β-sandwich structure, with strands β1, β5, β6, and β8 in one sheet and strands β2, β3, β4, and β7 in the other (Figure 1A). The overall structure of the HAUSP TRAF-like domain closely resembles the TRAF-C domain of the TRAF family of proteins [32–36]. In addition, the HAUSP TRAF-like domain contains a shallow surface groove in the middle of the β-sandwich (Figure 1A), which corresponds to the region where the receptor peptides bind [32–36].

**Figure 1:**
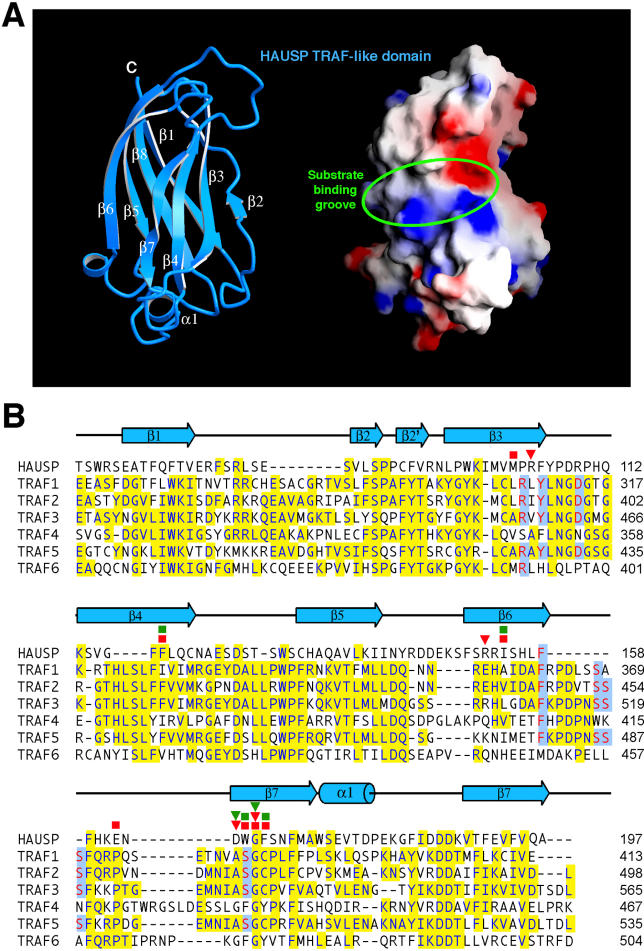
Structure of the HAUSP N-Terminal TRAF-Like Domain (A) Structure of the HAUSP TRAF-like domain in a ribbon diagram (left) and a surface representation (right). Secondary structural elements (left) and the putative substrate-binding groove (right) are labeled. (B) Sequence alignment of the HAUSP TRAF-like domain with other TRAF family members. Conserved residues are shown in yellow. Residues that interact with p53 through hydrogen bonds and van der Waals contacts are identified by green arrow heads and green squares, respectively. Residues that interact with MDM2 through hydrogen bonds and van der Waals contacts are indicated by red arrow heads and red squares, respectively. Conserved residues that are involved in binding to peptides in other TRAF family proteins, but not in HAUSP, are colored red and indicated by purple background.

**Table 1:**
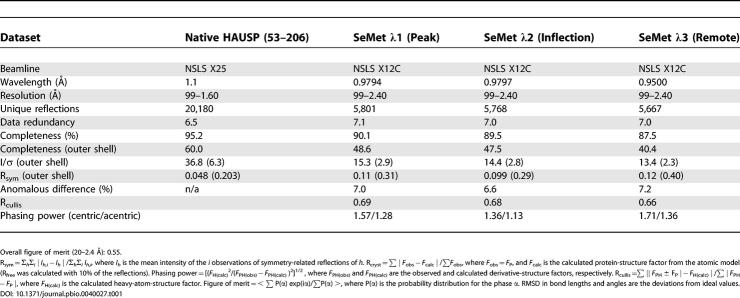
Summary of Crystallographic Analysis on HAUSP TRAF-Like Domain

**Table 2:**
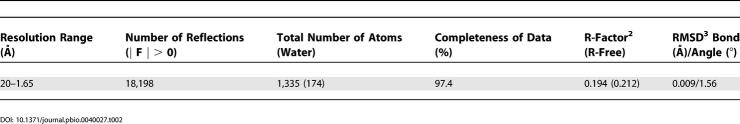
Refinement Statistics on the HAUSP TRAF-Like Domain

**Table 3:**
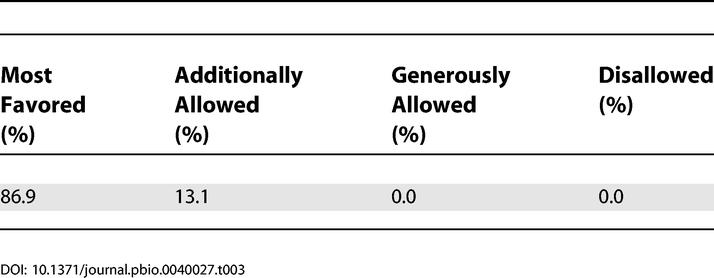
Ramachandran Plot of the Final Coordinates on the HAUSP TRAF-Like Domain

Despite overall structural similarity, amino acid composition in the putative substrate-binding groove of HAUSP differs significantly from that in TRAF2 (Figure 1B). For example, a set of peptide-interacting residues in TRAF2, including Arg393, Tyr395, Asp399, Phe447, Ser453, Ser454, Ser455, and Ser467, are highly conserved among TRAF1, 2, 3, and 5 [32–36]. However, most of these conserved residues are no longer retained in the HAUSP TRAF-like domain, suggesting different peptide-binding specificity. Moreover, the residues mediating peptide recognition in TRAF6, Arg392, Phe471, and Tyr473, are also missing in the HAUSP N-terminal domain structure [34] (Figure 1B). These observations indicate that the HAUSP TRAF-like domain may represent a new type of peptide-binding motif in the TRAF family.

### Structural Basis of p53 Recognition by HAUSP

We previously showed that the HAUSP TRAF-like domain stably interacts with a C-terminal peptide fragment (residues 357–382) of p53 and that deletion of 11 amino acids (residues 357–367) in p53 resulted in a complete loss of interaction with HAUSP [29]. Using an in vitro interaction assay, we performed mutagenesis to further define the HAUSP-binding element in p53 (Figure 2A). All mutant p53 fragments were individually purified to homogeneity and were examined for binding to HAUSP using gel filtration. The results are summarized in Figure 2A. The wild-type p53 fragment (residues 325–363), which exists as a constitutive tetramer in solution, formed a stable complex with HAUSP. Mutations of residues 357, 358, and 363 did not affect the ability of p53 (residues 325–367) to interact with HAUSP.

**Figure 2:**
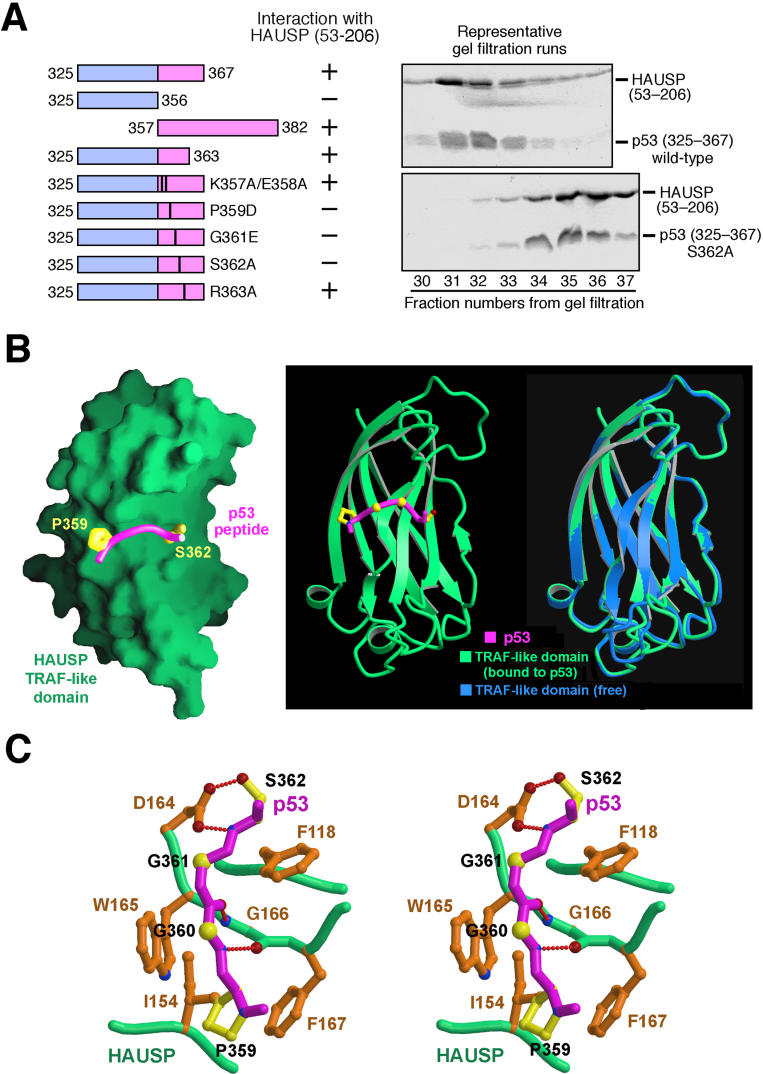
Structural Basis of p53 Recognition by HAUSP (A) Identification of a short-peptide fragment in p53 (residues 359–362) as the HAUSP-binding element. Various p53 fragments were individually incubated with HAUSP protein (residues 53–206) and their interactions were examined by gel filtration. The results are summarized in the left panel. Two representative experiments, for WT p53 (residues 325–367, WT) and mutant p53 (residues 325–367, S362A), are shown on the right panel. Note that, because the elution volumes for free HAUSP (residues 53–206) and for free p53 (residues 325–367) correspond to fraction numbers 36 and 35, these two proteins appear to co-migrate on gel filtration—but they do not interact with each other. The protein concentrations for gel filtration were: HAUSP (53–206), 55 μM; p53 WT, 46 μM; p53 mutant, 51 μM. (B) Overall structure of the HAUSP TRAF-like domain bound to p53 peptide is shown in a surface representation (left) and in a ribbon diagram (right). Binding by the p53 peptide does not induce any significant conformational changes in HAUSP as shown by the structural comparison of the free and p53-bound TRAF-like domain (right panel). (C) A stereo view of the specific interactions between p53 and HAUSP. Hydrogen bonds are represented by red dashed lines. All interacting residues are labeled.

In contrast, mutation of Pro359, Gly361, or Ser362 in p53 (residues 325–367) resulted in abrogation of its interaction with the HAUSP TRAF-like domain (Figure 2A). For example, while the wild-type p53 (residues 325–367) formed a stable complex with HAUSP (residues 53–206) as judged by their co-elution on gel filtration (Figure 2A, upper-right panel), the mutant p53 (S362A) and HAUSP (residues 53–206) did not interact with each other (Figure 2A, lower-right panel). It should be noted that, because the elution volume for HAUSP (residues 53–206) alone is very similar to that of the tetrameric p53 (residues 325–367) alone, the tetrameric mutant p53 (S362A) and HAUSP (residues 53–206) were present in similar fractions from gel filtration despite lack of interactions (Figure 2A, lower-right panel). Together, these observations suggest that the minimal HAUSP-binding element is within amino acids 359–363 of p53. Interestingly, calibration of the gel filtration column suggests that only one molecule of the HAUSP TRAF-like domain (18 kDa) is likely to be bound to a tetramer (19.6 kDa) of the p53 fragment (residues 325–367), as the complex was eluted with an apparent molecular weight of approximately 39 kDa. The precise stoichiometry between HAUSP and p53 should be determined by additional conclusive experimental approaches.

To elucidate the mechanism by which p53 recognizes HAUSP, we launched rigorous trials aimed at crystallizing the HAUSP TRAF-like domain bound to a synthetic p53 peptide. However, such effort did not yield crystals that are suitable for X-ray-diffraction studies. To facilitate formation of a stable p53–HAUSP complex, we generated a chimeric protein with the C-terminus of the HAUSP TRAF-like domain (residues 53–199) fused to ten amino acids corresponding to p53 residues 359–368. We reasoned that such design would ensure a 1:1 stoichiometry between HAUSP and the p53 peptide. Indeed this engineering effort proved effective, and diffraction-quality crystals were successfully obtained. The structure of the HAUSP TRAF-like domain bound to p53 (residues 359–368) was determined at 2.3-Å resolution by molecular replacement (Table 4; Figure 2B).

**Table 4:**
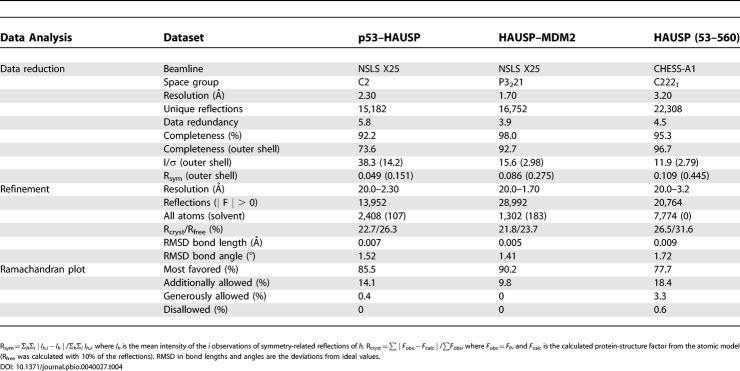
Summary of Crystallographic Analysis

The p53 peptide binds to the shallow surface groove near one edge of the β-sandwich in the TRAF-like domain (Figure 2B). Only four contiguous amino acids of p53, Pro359-Gly360-Gly361-Ser362, make specific interactions to residues in HAUSP, whereas Arg363 adopts a well-defined conformation but is not directly involved in HAUSP recognition (Figure 2C). At the N-terminus, the side chain of Pro359 is nestled in a hydrophobic pocket at the edge of the β-sandwich, which is formed by the backbone Cα atom of Gly166 and the hydrophobic side chains of Phe167, Trp165, and Ile154 (Figure 2C). At the C-terminus, the carboxylate side chain of Asp164 accepts two hydrogen bonds from the amide and the hydroxyl groups of Ser362. In addition, the side chain of Ser362 makes van der Waals contacts to Phe118. In the center of the bound p53 peptide, the amide and carbonyl groups of Gly360 are hydrogen-bonded to the main-chain groups of Gly166 from HAUSP (Figure 2C). The main-chain Cα of Gly360 makes van der Waals contacts to Trp165 in HAUSP. Residues 364–368 of p53 are disordered in the crystals and do not contribute to HAUSP binding. A previous report identified a number of amino acid residues in the HAUSP TRAF-like domain that may play important roles in binding to p53 [30]. Our structure shows how the majority of these residues interact with specific residues in p53.

The structural observations are fully consistent with our mutagenesis data (Figure 2A and 2C). For example, Pro359 and Ser362 in the p53 peptide anchor its interaction with the HAUSP TRAF-like domain (Figure 2C); missense mutation of Pro359 or Ser362 in p53 abolished its binding to HAUSP (Figure 2A). To further corroborate the structural findings, we also performed additional mutagenesis on the HAUSP TRAF-like domain. Mutation of Trp165 and Phe167 in HAUSP, both involved in the hydrophobic pocket that accommodates Pro359 of p53, resulted in a complete loss of binding to p53 (unpublished data). Similarly, mutation of Asp164, which forms hydrogen bonds with S362 of p53, also significantly weakened the ability of HAUSP to bind to p53 (unpublished data).

### Mapping HAUSP–MDM2 Interactions

MDM2 is known to specifically associate with HAUSP and represents a physiological substrate for HAUSP-mediated deubiquitylating activity. To elucidate the mechanism by which HAUSP recognizes MDM2, we first sought to identify the minimal domain in HAUSP that is responsible for binding to MDM2, and the results are summarized in Figure 3A. To our surprise, the N-terminal TRAF-like domain of HAUSP (residues 1–206) was found to form a stable complex with MDM2 (residues 170–423). This result indicates that the same domain of HAUSP that interacts with p53 is also responsible for binding to MDM2. Neither the central isopeptidase domain nor the C-terminal extension of HAUSP was required for binding to MDM2 (residues 170–423) (Figure 3A).

**Figure 3:**
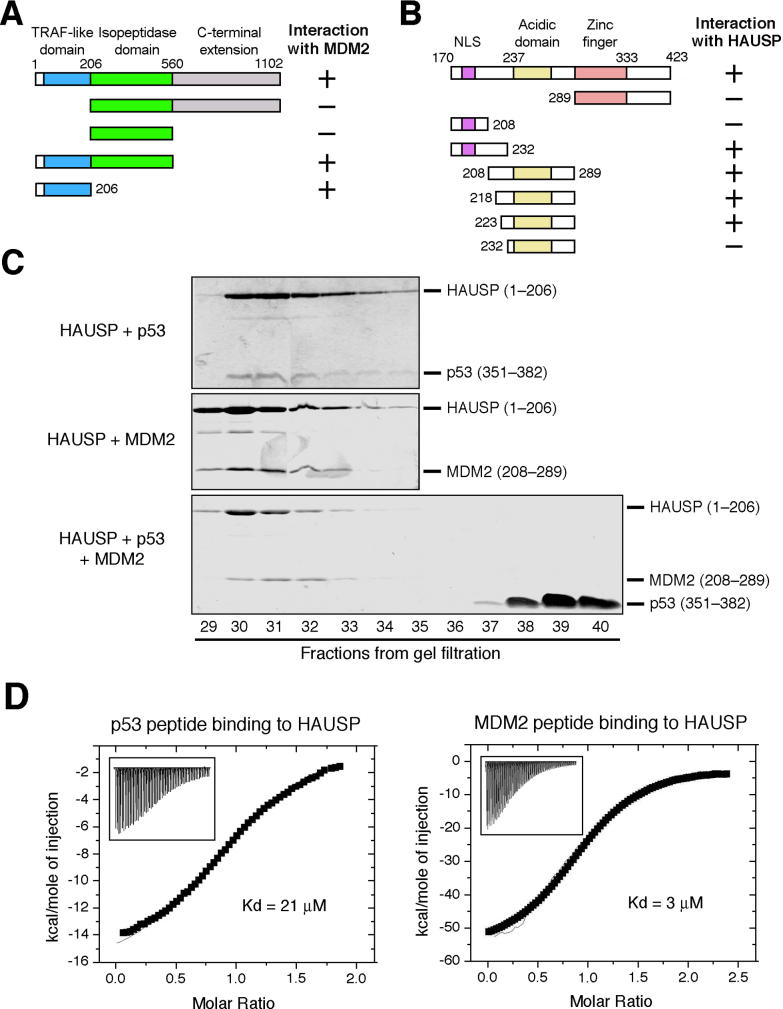
HAUSP Preferentially Forms a Stable HAUSP–MDM2 Complex in the Presence of Excess p53 (A) The TRAF-like domain of HAUSP is responsible for binding to MDM2. Various HAUSP fragments were individually incubated with MDM2 protein (residues 170–423) and their interactions were examined by gel filtration. The results are summarized here. (B) Identification of a minimal HAUSP-binding element in MDM2. Various MDM2 fragments were individually incubated with HAUSP TRAF-like domain (residues 53–206) and their interactions were examined by gel filtration. The results are summarized here. (C) HAUSP preferentially forms a stable HAUSP–MDM2 complex in the presence of excess p53. HAUSP (residues 1–206) interacts with both p53 (residues 351–382, upper panel) and MDM2 (residues 208–289, middle panel). However, in the presence of a 10-fold excess amount of p53, HAUSP formed a stable complex only with MDM2 (lower panel). The relevant peak fractions were visualized by SDS-PAGE followed by Coomassie staining. (D) Determination of binding affinities between the HAUSP TRAF-like domain (residues 53–206) and peptides derived from p53 and MDM2 by ITC. The p53 and MDM2 peptides contain residues 351–382 and 208–242, respectively. The binding affinities for the p53 and MDM2 peptides are 3 and 21 μM, respectively.

Next, we mapped the minimal HAUSP-binding element in MDM2, and the results are summarized in Figure 3B. The full-length human MDM2 (known as HDM2), containing 491 amino acids [37], can be divided into four major conserved regions: an N-terminal domain responsible for binding to p53, a highly acidic region that is involved in binding to multiple proteins, a putative Cys4-type zinc-finger domain, and a RING-finger domain responsible for the E3 ubiquitin ligase activity of MDM2 [37]. We first found that a 33-kDa fragment of MDM2 (residues 170–432) formed a stable complex with the full-length HAUSP as well as with the HAUSP N-terminal TRAF-like domain. Next, we generated a series of deletion variants of MDM2 and assayed their interactions with the HAUSP TRAF-like domain using a gel filtration assay. Neither the central acidic region nor the zinc-finger domain of MDM2 interacted with HAUSP (residues 53–206). An MDM2 fragment encompassing residues 223–289 or residues 170–232 formed a stable complex with the HAUSP TRAF-like domain (residues 53–206), suggesting an essential role of amino acids 223–232 in MDM2 (Figure 3B). Supporting this notion, the ten-amino acid MDM2 fragment (residues 223–232) was found to form a stable complex with the HAUSP TRAF-like domain (unpublished data).

### Mutually Exclusive HAUSP Binding by p53 and by MDM2

The fact that both a p53 C-terminal peptide fragment and a short-peptide sequence preceding the acidic domain of MDM2 bind to the N-terminal TRAF-like domain of HAUSP raised an intriguing possibility: HAUSP binding by p53 and by MDM2 might be mutually exclusive. To test this hypothesis, we performed an in vitro competition assay using gel filtration chromatography. The HAUSP TRAF-like domain (residues 1–206) was incubated with one molar equivalence of MDM2 (residues 208–289) in the presence of 10-fold molar equivalence of p53 (residues 351–382), and then the mixture was subjected to a gel filtration analysis. Despite a 10-fold excess of the p53 peptide over MDM2, the MDM2 fragment, but not the p53 peptide, formed a stable complex with HAUSP (residues 1–206) (Figure 3C). This result suggests that MDM2 binds to HAUSP with a higher affinity than does p53.

To assess the strength of HAUSP binding by p53 and by MDM2, we measured the binding affinity between the HAUSP TRAF-like domain (residues 53–206) and a p53 peptide (residues 351–382) or an MDM2 peptide (residues 208–242) using isothermal titration calorimetry (ITC). Under identical experimental conditions, the MDM2 peptide bound to the HAUSP TRAF-like domain with a dissociation constant of 3 μM, 7-fold tighter than that of the p53 peptide (Figure 3D). Thus these experiments confirm the competitive binding edge of MDM2 over p53. The observed binding affinity between the p53 peptide and the HAUSP TRAF-like domain is in agreement with a previous report [30]. It should be noted that the binding affinity of the full-length MDM2 is likely to be higher than the value reported here, because the full-length MDM2 protein contains at least two more binding sites for HAUSP (see Discussion), and the multiple binding sites serve to significantly increase the effective protein concentration.

### Structural Basis of HAUSP Recognition by MDM2

Intriguingly, the minimal MDM2 fragment (residues 223–232) required for binding to HAUSP exhibited little sequence homology to the p53 fragment that interacts with the same domain of HAUSP. To elucidate the mechanism by which MDM2 recognizes HAUSP, we sought to determine the structure of HAUSP TRAF-like domain bound to the minimal MDM2 fragment. After many unsuccessful trials, we once again succeeded by engineering a chimeric protein with the MDM2 peptide (residues 223–232) fused to the C-terminus of the HAUSP TRAF-like domain (residues 53–197). We crystallized this chimeric protein and solved its structure at 1.7-Å resolution (Table 4 and Figure 4A).

**Figure 4:**
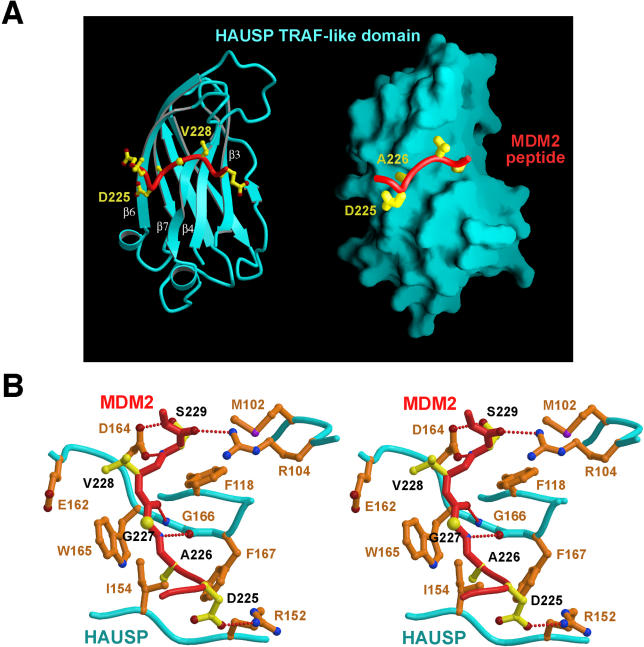
Structural Basis of MDM2 Recognition by HAUSP (A) Overall structure of the HAUSP TRAF-like domain bound to MDM2 peptide is shown in a ribbon diagram (left) and in a surface representation (right). The important MDM2 residues are highlighted in yellow. (B) A stereo view of the specific interactions between MDM2 and HAUSP. These interactions are more extensive than those between p53 and HAUSP. Hydrogen bonds are represented by red dashed lines. All interacting residues are labeled.

In the structure of the HAUSP–MDM2 fusion protein, the MDM2 peptide (residues 223–230) is bound to the shallow surface groove on one side of the β-sandwich in the TRAF-like domain, the same site where p53 binds. The MDM2 peptide assumes an extended main-chain conformation, extending across strands β3, β4, β6, and β7 (Figure 4A). Five residues in the MDM2 peptide, Asp225-Ala226-Gly227-Val228-Ser229, make extensive polar and non-polar interactions with the surface-binding residues from all four strands, particularly strand β7 (Figure 4B). Although Asp223, Leu224, and Glu230 of MDM2 adopt well-defined conformation in the structure, they do not make a direct contribution to interactions with HAUSP.

At the N-terminal end of the MDM2 peptide, the carboxylate side chain of Asp225 accepts a charge-stabilized hydrogen bond from the guanidinium group of Arg152 in HAUSP (Figure 4B). The main-chain conformation of Asp225 is stabilized by a pair of intra-molecular hydrogen bonds between the carbonyl group of Asp223 and the amide groups of Asp225 and Ala226. In the center of the MDM2 peptide, three residues make a number of van der Waals interactions to HAUSP. The side chain of Ala226 in MDM2 packs against a hydrophobic pocket formed by the backbone Cα atom of Gly166 and the side chains of Phe167, Trp165, and Ile154 in HAUSP. The main-chain groups of Gly227 in MDM2 make two hydrogen bonds to Gly166 in HAUSP, whereas the backbone Cα atom of Gly227 is within van der Waals–contact distance of the hydrophobic side chain of Trp165 in HAUSP. The side chain of Val228 in MDM2 interacts with a hydrophobic-surface patch formed by the side chain of Trp165 and the aliphatic portion of the side chains of Glu162 and Asp164 in HAUSP. At the C-terminal end of the MDM2 peptide, Ser229 in MDM2 makes a total of three hydrogen bonds to surface residues in HAUSP. The amide and the hydroxyl groups of Ser229 donate two hydrogen bonds to the carboxylate side chain of Asp164 in HAUSP, and the carbonyl group of Ser229 accepts a hydrogen bond from the guanidinium group of Arg104 in HAUSP (Figure 4B).

### Comparison of HAUSP Binding by MDM2 and by p53

To compare the specific recognition of p53 and MDM2 by HAUSP, we superimposed the structure of the HAUSP TRAF-like domain bound to p53 peptide to that bound to MDM2. This alignment resulted in a root-mean-square deviation (RMSD) of 0.37 Å for 134 aligned-backbone Cα atoms, with the bound p53 and MDM2 peptides well superimposed to each other (Figure 5A).

**Figure 5:**
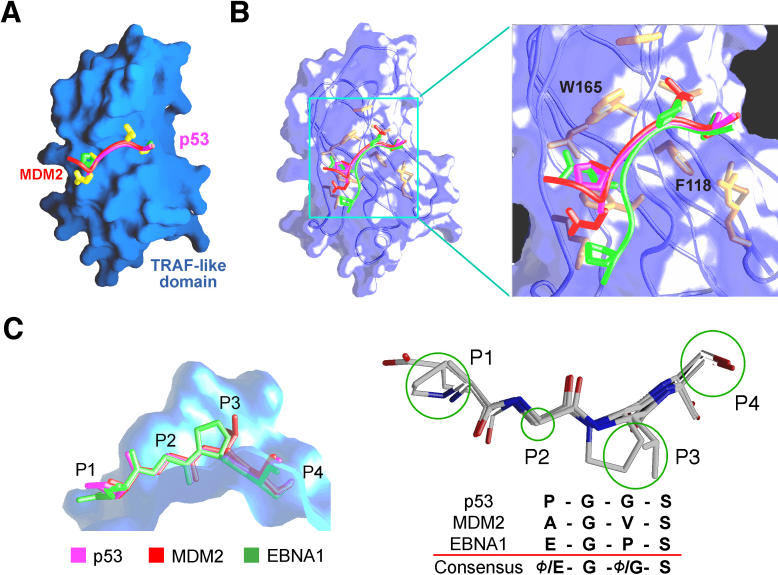
Structural Comparison of Peptide Binding by HAUSP Reveals a Consensus Sequence (A) MDM2 peptide (red) binds to the same surface groove as the p53 peptide (magenta). Residues from MDM2 and p53 are shown in yellow and green, respectively. (B) Superposition of three HAUSP-binding peptides derived from MDM2 (red), p53 (magenta), and EBNA1 (green). The HAUSP TRAF-like domain is shown in a transparent surface representation, with critical residues shown in brown. (C) Structural alignment of HAUSP-binding peptides reveals a consensus sequence. The HAUSP surface groove (in a transparent surface representation) for binding to the consensus tetrapeptide is shown in the left panel. The consensus sequence is shown in the right panel.

There are a number of common themes for the recognition of p53 and MDM2 by HAUSP. Both p53 and MDM2 peptides bind to the same surface groove in the HAUSP TRAF-like domain, sharing similar overall orientation (Figure 5A and 5B). Two non-polar residues, Pro359 in p53 and Ala226 in MDM2, are positioned at the same site upon binding to HAUSP and make similar van der Waals contacts with a hydrophobic-surface pocket near the edge of β-sandwich in HAUSP. The residues following Pro359 in p53 and Ala226 in MDM2 are both glycines, which are very well superimposed and make conserved main-chain hydrogen bonds to Gly166 in the β7 strand of HAUSP (Figure 5B and 5C). Another conserved residue in both peptides is Ser362 in p53 and Ser229 in MDM2, which in both cases make hydrogen bonds to Asp164 in HAUSP and make van der Waals contacts to Phe118 in HAUSP. Considering the multiple specific interactions between this Ser residue and residues from HAUSP, it is likely that this Ser plays an anchoring role in the specific recognition of both p53 and MDM2 by HAUSP.

Despite these common features, important differences exist for HAUSP binding by p53 and by MDM2, which explain the competitive edge of MDM2 over p53. Gly361 in p53, which does not directly interact with HAUSP, is substituted by Val228 in MDM2, resulting in additional van der Waals contacts with Trp165 and Glu162 in HAUSP (Figure 4B). Perhaps more importantly, Asp225 in MDM2 makes a direct hydrogen bond to Arg152 and a water-mediated hydrogen bond to Ser168 in HAUSP; yet such interactions are absent in the case of p53. Compared to p53, the more extensive interactions between MDM2 and HAUSP provide a mechanistic explanation to our biochemical observation that MDM2 out-competed excess p53 for binding to HAUSP.

### Peptide Recognition by HAUSP

In this paper, we report the structural basis for the differential recognition of p53 and MDM2 by HAUSP. HAUSP was previously known to bind to another protein named EBNA1 [38,39], and the crystal structure of the HAUSP TRAF-like domain bound to a peptide derived from EBNA1 was recently reported [30]. The amino acid sequences of the three HAUSP-binding peptides, PGGSR in p53, DAGVSE in MDM2, and PGEGPS in EBNA1, share no obvious sequence homology with each other; however, they all bind to the same surface groove on the TRAF-like domain of HAUSP (Figure 5B). Comparison of these three structures reveals how HAUSP specifically recognizes all three distinct peptides.

Structure-based sequence alignment of the three HAUSP-binding peptides revealed a conserved four-residue binding motif with the consensus sequence of (Ø/E)-G-(Ø/G)-S, where Ø denotes a non-polar residue (Figure 5C). In all three structures, the four-residue motif, in an extended conformation, binds to the same substrate-recognition site on HAUSP (Figure 5C) and makes conserved contacts with the protein (Figure 5B). The first residue, P1, in the binding motif is either a non-polar residue such as Pro359 in p53, Ala226 in MDM2, or Glu444 in EBNA1, which also has an aliphatic portion in its side chain (Figure 5C). P1 makes van der Waals interactions with a hydrophobic-surface pocket on one edge of the β-sandwich in HAUSP. P2, an invariant Gly in all three peptides, forms two main-chain hydrogen bonds with Gly166 in the β7 strand of HAUSP. P3, a hydrophobic residue in MDM2 and EBNA1, stacks against a small hydrophobic-surface patch on HAUSP. This residue is Gly in p53, resulting in a loss of van der Waals interaction at the P3 position and consequently contributing to a relatively low binding affinity [38] (Figure 3C). P4, an invariant Ser in all three peptides, makes two hydrogen bonds to the carboxylate side chain of Asp164 in HAUSP and contacts Phe118 from the β4 strand of HAUSP. This analysis identifies Ser at the P4 position as the anchoring residue for specific binding of peptides to HAUSP.

The residues outside the central HAUSP-binding motif are diverse, both in terms of their corresponding positions and in terms of the interactions that they mediate (Figure 5B). Both MDM2 and EBNA1, but not p53, contain extra HAUSP-interacting residues at the N-terminal end of the motif. In EBNA1 peptide, the N-terminal Pro442 and Gly443 run parallel to the edge of the HAUSP β7 strand, forming hydrogen bonds between the carbonyl group of Asp441 and the side chain of Asn169 in HAUSP and between the carbonyl group of EBNA1 Pro442 and the amide group of Ser168 in HAUSP [30]. Moreover, Pro442 has additional hydrophobic contacts with Phe167 in HAUSP. These additional interactions are very different from those between MDM2 and HAUSP (Figure 4B) but explain why EBNA1 out-competes p53 for binding to HAUSP [30].

The fact that most of the conserved peptide-binding residues in TRAFs are no longer present in the structure of the HAUSP TRAF-like domain (Figure 1B) indicates that the TRAF-like domain of HAUSP represents a new peptide-binding motif in the TRAF family. Supporting this notion, the minimal HAUSP-binding motif does not match any of the known TRAF-binding sequences, including the major consensus sequence of (P/S/A/T)X(Q/E)E and the minor consensus sequence of PXQXXD for TRAF1, 2, 3, and 5 [33,36], and the TRAF6-binding motif of PxExx(¢/acidic) [34].

### Coordination of Substrate Recognition and Deubiquitylation by HAUSP

HAUSP is a representative member of the UBP family of DUBs, which are known to recognize specific substrates for deubiquitylation. The recognition of a substrate is expected to be coupled to its deubiquitylation by HAUSP. However, there is a complete lack of understanding for this process at the present time, despite the fact that the structure of the catalytic core domain of HAUSP has already been determined [29]. To investigate this process, we sought to determine the crystal structure of a larger HAUSP fragment (residues 53–560), which contains both the substrate-binding TRAF-like domain and the catalytic core domain. We succeeded in the generation of crystals for this HAUSP fragment and solved its structure at 3.2 Å-resolution (Table 4). The structure contains two well-ordered domains, the TRAF-like domain as a β-sandwich and the isopeptidase domain as a tripartite architecture (Figure 6A). Interestingly, there is very limited interaction between these two domains, and the intervening linker sequences (residues 199–206) have high-temperature factors in the crystals, suggesting that the substrate-binding domain is coupled to the catalytic domain through a relatively flexible linker sequence. In addition, the ubiquitin-binding pocket of the catalytic core domain and the substrate-binding groove of the TRAF-like domain are located on the same side of the molecule (Figure 3).

**Figure 6:**
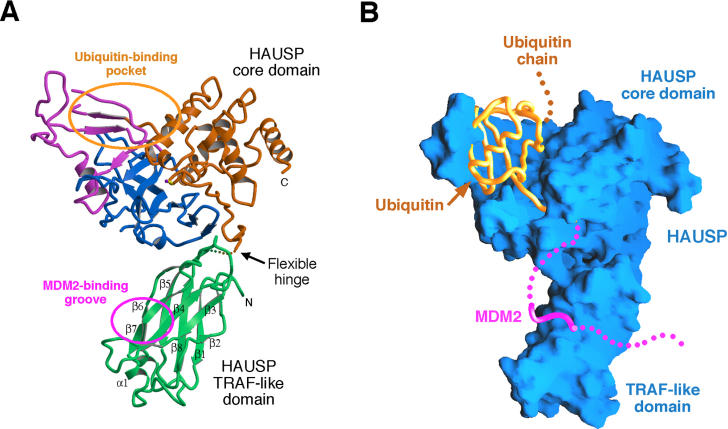
Structure of an Extended HAUSP Fragment (A) Structure of a HAUSP fragment (residues 53–560) that contains both the substrate-binding (green) and the catalytic domains. Binding sites for ubiquitin and substrate are indicated. The linker sequences between these two domains have high-temperature factors and are flexible in the crystals. (B) A structure-based model showing HAUSP bound to an ubiquitylated MDM2. Only one ubiquitin moiety and the MDM2 peptide are shown in this model.

On the basis of the structure and previous information about ubiquitin binding [29], we modeled how HAUSP may recognize an ubiquitylated MDM2 molecule (Figure 6B). In this model, a small peptide region (residues 225–230) of MDM2 anchors its binding to the TRAF-like domain of HAUSP. A conjugated ubiquitin moiety is bound by the Fingers domain of HAUSP, with the C-terminus of ubiquitin docked in the active-site cleft between the Palm and the Thumb (Figure 6B). The close proximity of the C-terminus of HAUSP-bound ubiquitin and the MDM2 peptide suggests that HAUSP likely prefers to cleave the ubiquitin chain at the proximal end of the ubiquitylated MDM2.

## Discussion

The critical role of HAUSP in regulating the p53–MDM2 pathway was first revealed by the finding that HAUSP can specifically recognize and deubiquitylate p53 both in vivo and in vitro [24]. However, the concept of HAUSP as a specific p53-stabilizing protein and candidate tumor suppressor was challenged by the recent findings that HAUSP also specifically recognizes MDM2 and stabilizes MDM2 through direct deubiquitylation [27,28]. Based on these findings, a dynamic role of HAUSP in the p53–MDM2 pathway was proposed [26]. However, the mechanisms by which HAUSP recognizes p53 and MDM2, as well as the exact role of HAUSP in the p53–MDM2 pathway, remained enigmatic.

In this study, we report four important and novel findings. First, the N-terminal TRAF-like domain of HAUSP/USP7 was identified to specifically recognize both p53 and MDM2. This finding also indicates that the substrate-recognition domain of a UBP (such as HAUSP) may target more than one cellular protein. Second, MDM2 binding to HAUSP was found to be mutually exclusive with p53 binding to HAUSP. The HAUSP-binding elements were mapped to a peptide fragment in the carboxy-terminus of p53 and to a short-peptide region preceding the acidic domain of MDM2. A minimal HAUSP-binding peptide derived from MDM2 efficiently displaced p53 from the p53–HAUSP complex in a competition assay and formed a stable HAUSP–MDM2 complex. Third, the molecular basis of HAUSP-mediated recognition of p53 and MDM2 was revealed by the high-resolution crystal structures of the HAUSP TRAF-like domain bound to peptides derived from p53 and MDM2. Structural comparison reveals that MDM2 recognizes the same surface groove in the HAUSP TRAF-like domain as that made by the p53 peptide. Compared to p53, MDM2 makes conserved, yet more extensive, contacts with HAUSP. Last, but not least, structural analysis reveals a consensus peptide sequence for HAUSP recognition. This consensus sequence greatly facilitates any effort aimed at identifying additional substrates for HAUSP/USP7. For example, Hdmx, an MDM2 homologue, was shown to bind to HAUSP [40]; examination of the primary sequences of Hdmx identified a consensus HAUSP-binding peptide, A470GAS473. Such analysis also identifies the TRAF-like domain in HAUSP as a new class of peptide-binding TRAF domain.

Our studies provide an important framework for understanding the function of HAUSP in the p53–MDM2 pathway. Although HAUSP can bind to p53, our competition data suggest that HAUSP exhibits a higher binding affinity for MDM2, which likely translates into a stronger preference for MDM2 deubiquitylation and stabilization. This conclusion further suggests that MDM2 may represent a better substrate for HAUSP activity under physiological conditions and argues for a critical role of HAUSP in antagonizing the auto-ubiquitylation function of MDM2. This analysis is fully consistent with the observation that p53 is stabilized but MDM2 is destabilized in HAUSP-ablated cells [26–28].

For a given protein such as MDM2 or p53, ubiquitylation frequently occurs to multiple Lys residues that are spread out in the primary sequences and in space. In this case, how can a UBP effectively deubiquitylate at multiple sites? The answer to this question is inferred from our current study. First, it is possible that a given protein may contain multiple binding sites for the corresponding UBP. Supporting this notion, a consensus HAUSP-binding element contains only four amino acids, (Ø/E)-G-(Ø/G)-S, which predicts a statistically reasonable chance for finding additional sites in the same protein. Indeed, an examination of the complete human MDM2 sequence revealed two additional candidate HAUSP-binding elements, D117SGTS121 and L129EGGS133. Thus the presence of multiple HAUSP-recognition sites in a given protein may greatly facilitate efficient deubiquitylation. Although p53 contains only one HAUSP-binding site, it forms a tetramer in cells and thus generates four potential binding sites for HAUSP. Interestingly, however, the tetrameric p53 fragment appeared to interact with only one HAUSP molecule (Figure 2A), presumably owing to structural constraint. Second, the linker sequence between the TRAF-like domain and the catalytic domain is flexible (Figure 6A), which allows some lateral movement between these two domains and hence enhanced access to multiple ubiquitylation sites within the same substrate.

It is worth noting that the consensus peptide sequence for HAUSP binding has Ser, which can be phosphorylated, as an anchoring residue. This residue makes a conserved hydrogen bond to the negatively charged Asp164 in p53 (Figure 2), in MDM2 (Figure 4), and in EBNA1 [30]. Structural analysis suggests that phosphorylation of this Ser residue may have a negative effect on its ability to contribute to HAUSP binding.

The interactions between HAUSP and p53 or MDM2 are likely to be more complex in vivo, not only due to the presence of multiple binding sites in MDM2 but also because of the oligomeric nature of p53 and possibly HAUSP. Although HAUSP was shown to be a homogeneous monomer in solution by analytical ultracentrifugation [38], recent data suggested that HAUSP may form a homo-dimer in cells [41]. Nonetheless, HAUSP was found to bind to p53 monomer and tetramer with similar binding affinities [38], suggesting that HAUSP may not exhibit a strong preference to any specific oligomeric form of p53. This notion is consistent with our biochemical and structural observations (Figure 2), which demonstrate that the HAUSP-binding element of p53 (residues 359–362) is located C-terminal to the tetramerization domain (residues 325–355).

Finally, our studies also have important ramifications for potential drug screening. Since HAUSP plays an essential role in the stabilization of MDM2, the negative regulation of HAUSP may lead to suppression of MDM2 activity, which in turns stabilizes the tumor-suppressor protein p53. Our current structural studies identify differential features for HAUSP binding by MDM2 and by p53. For example, MDM2 contains an additional binding residue (Asp225) that makes contacts to an area of HAUSP that is irrelevant for p53 binding (Figure 5B). Strategies can be devised to screen for specific inhibitors that target this interface, thus destabilizing HAUSP–MDM2 interactions. Additionally, because complete inhibition of HAUSP is likely to have a far greater negative effect on MDM2 over p53, the entire surface groove of the HAUSP TRAF-like domain can be explored for inhibitor design and screening. The available high-resolution structures should greatly facilitate this process. Such effort can be carried out in conjunction with inhibitor screening aimed at suppressing the isopeptidase activity of HAUSP.

## Materials and Methods

### Protein preparation

All constructs were generated using a standard PCR-based cloning strategy. The HAUSP TRAF-like domain (residues 53–206) and the HAUSP long fragment (residues 53–560) were cloned into pGEX-2T (Pharmacia-LKB Technology, Uppsala, Sweden), and were overexpressed in *Escherichia coli* strain BL21(DE3) as N-terminally GST-tagged proteins. Seleno-Met-substituted HAUSP TRAF-like domain (residues 53–206) was expressed in *E. coli* B834(DE3) (Novagen, Madison, Wisconsin, United States) in M9 minimal medium supplemented with 50 mg/l of selenomethionine. To generate the p53–HAUSP chimeric protein, the complementary DNA sequence encoding the p53 peptide (residues 359–368) was inserted into the sequence of the 3′ primer used for the PCR reaction of the HAUSP TRAF-like domain (residues 53–199). A similar strategy was adopted to generate the HAUSP–MDM2 chimeric protein, in which the MDM2 peptide (residues 223–232) was fused to the C-terminus of the HAUSP N-terminal domain (residues 53–197). Both chimeric proteins were cloned into the vector pGEX-2T (Pharmacia-LKB Technology) and were overexpressed in BL21(DE3). All proteins were purified to homogeneity as previously described [29].

### Crystallization and structure determination of the HAUSP TRAF-like domain

Crystals were grown by the hanging-drop vapor-diffusion method by mixing the HAUSP protein (residues 53–206) (∼25 mg/ml) with an equal volume of reservoir solution containing 100 mM Tris (pH 8.0), 240 mM CaCl2, 20 mM (NH4)2SO4, and 6.5% PEG4000 (w/v), at 12 °C. Small crystals appeared after 2–3 d and continued to grow to full size in 3–4 wk. The crystals belong to the space group P212121 and contain one molecule per asymmetric unit. The unit cell has a dimension of a = 45.17 Å, b = 52.24 Å, and c = 63.65 Å. Crystals were equilibrated in a cryoprotectant buffer containing reservoir buffer plus 25% glycerol (v/v) and were flash frozen in a cold nitrogen stream at −170 °C. The native and multiple anomalous dispersion (MAD) datasets were collected at National Synchrotron Light Source (NSLS) beamlines X25 and X12C, respectively. All X-ray-diffraction data described in this paper were processed using the software Denzo and Scalepack [42]. The structure was determined by MAD, using SOLVE [43], and the selenium sites were refined using MLPHARE [44]. All atomic models described in this study were built using O [45] and were refined using CNS [46].

### Crystallization and structure determination of the p53–HAUSP chimeric protein

Crystals were grown by the hanging-drop vapor-diffusion method by mixing the protein (∼20 mg/ml) with an equal volume of reservoir solution containing 100 mM Tris (pH 7.5), 200 mM CaCl2, and 9% PEG2000 monomethylether (w/v). The crystals belong to the space group C2, and contain two molecules per asymmetric unit. The unit cell has a dimension of a = 87.76 Å, b = 39.56 Å, and c = 101.9 Å, and β = 105.3, and contains two molecules per asymmetric unit. The native dataset was collected at NSLS X25. The structure was determined by molecular replacement using AMoRe [47] and was refined at 2.3-Å resolution.

### Crystallization and structure determination of the HAUSP–MDM2 chimeric protein

Crystals were grown by the hanging-drop vapor-diffusion method by mixing the protein (∼15 mg/ml) with an equal volume of reservoir solution containing 100 mM Tris (pH 8.5), 300 mM CaCl2, and 26% PEG4000 (w/v). The crystals belong to the space group P3221 and contain one molecule per asymmetric unit. The unit cell has a dimension of a = 37.53 Å, b = 37.53 Å, and c = 177.3 Å. The native dataset was collected at NSLS X25. The structure was determined by molecular replacement using AMoRe [47] and was refined at 1.7-Å resolution.

### Crystallization and structure determination of HAUSP (53–560)

Crystals were grown by the hanging-drop vapor-diffusion method by mixing the protein (∼15 mg/ml) with an equal volume of reservoir solution containing 100 mM phosphate–citrate (pH 4.2), 0.8% PEG10,000, and 50 mM 1,6-Hexandiol. Small crystals appeared after 2 d and were used as seeds to generate larger crystals. The crystals belong to the space group C2221, and contain two molecules per asymmetric unit. The unit cell has a dimension of a = 97.63 Å, b = 219.9 Å, and c = 130.5 Å. The native dataset was collected at CHESS-A1. The structure was determined by molecular replacement using AMoRe [47] and was refined at 3.2-Å resolution.

### In vitro binding assays using gel filtration

Size-exclusion chromatography (gel filtration), using a Superdex-200 column (10/30, Amersham Pharmacia Biotech, Piscataway, New Jersey, United States), was employed to carry out all the in vitro binding assays. Proteins subjected to binding tests were incubated at 4 °C for at least 45 min to allow equilibrium to be reached. The flow rate was 0.5 ml/min, and the buffer contained 25 mM Tris (pH 8.0), 100 mM NaCl, and 2 mM DTT. All fractions were collected at 0.5 ml each. Aliquots of relevant fractions were mixed with SDS sample buffer and subjected to SDS-polyacrylamide gel electrophoresis. The proteins were visualized by Coomassie Blue staining. The column was calibrated with molecular-mass standards. For the competition experiment (Figure 3C), 10-fold molar equivalence of the p53 peptide was first incubated with the HAUSP TRAF-like domain for 5 min at 4 °C, then the complex was further incubated with one molar equivalence of MDM2 peptide for 45 min at 4 °C before the mixture was subjected to gel filtration analysis.

### ITC

HAUSP N-terminal domain (residues 53–206), the p53 (residues 351–382) peptide, and the MDM2 (residues 208–242) peptide were prepared in the buffer containing 10 mM HEPES (pH 8.0) and 100 mM NaCl. The Micro Calorimetry System (Microcal, Amherst, Massachusetts, United States) was used to perform the ITC measurements for the interaction between the HAUSP N-terminal domain and the peptides. The titration data were collected at 4 °C and were analyzed using the ORIGIN data-analysis software (Microcal Software, Northampton, Massachusetts, United States).

## Supporting Information

### Accession Numbers

The Protein Data Bank (http://www.rcsb.org/pdb) accession numbers for the atomic coordinates of the HAUSP TRAF-like domain alone, the HAUSP TRAF-like domain bound to p53 and MDM2 peptides, and HAUSP (residues 53–560) are 2F1W, 2F1X, 2F1Y, and 2F1Z, respectively.

## Abbreviations

DUB: deubiquitylating enzyme
HAUSP: herpesvirus-associated ubiquitin-specific protease (also known as USP7)
ITC: isothermal titration calorimetry
MAD: multiple anomalous dispersion
NSLS: National Synchrotron Light Source
RMSD: root-mean-square deviation
TRAF: tumor necrosis factor–receptor associated factor
UBP: ubiquitin-specific processing protease
